# IoTMindCare: An Integrative Reference Architecture for Safe and Personalized IoT-Based Depression Management

**DOI:** 10.3390/s25226994

**Published:** 2025-11-15

**Authors:** Sanaz Zamani, Roopak Sinha, Samaneh Madanian, Minh Nguyen

**Affiliations:** 1 Department of Computer Science and Software Engineering, Auckland University of Technology, Auckland 1010, New Zealand; sanaz.zamani@autuni.ac.nz (S.Z.); minh.nguyen@aut.ac.nz (M.N.); 2 School of Information Technology, Deakin University, Burwood, VIC 3125, Australia; 3 Department of Data Science and Artificial Intelligence, Auckland University of Technology, Auckland 1010, New Zealand; sam.madanian@aut.ac.nz

**Keywords:** Internet of Things, smart home, system architecture, health safety, personalized health, depression management

## Abstract

Depression affects millions of people worldwide. Traditional management relies heavily on periodic clinical assessments and self-reports, which lack real-time responsiveness and personalization. Despite numerous research prototypes exploring Internet of Things (IoT)-based mental health support, almost none have translated into practical, mainstream solutions. This gap stems from several interrelated challenges, including the absence of robust, flexible, and safe architectural frameworks; the diversity of IoT device ownership; the need for further research across many aspects of technology-based depression support; highly individualized user needs; and ongoing concerns regarding safety and personalization. We aim to develop a reference architecture for IoT-based safe and personalized depression management. We introduce *IoTMindCare*, integrating current best practices while maintaining the flexibility required to incorporate future research and technology innovations. A structured review of contemporary IoT-based solutions for depression management is used to establish their strengths, limitations, and gaps. Then, following the Attribute-Driven Design (ADD) method, we design *IoTMindCare*. The Architecture Trade-off Analysis Method (ATAM) is used to evaluate the proposed reference architecture. The proposed reference architecture features a modular, layered logical view design with cross-layer interactions, incorporating expert input to define system components, data flows, and user requirements. Personalization features, including continuous, context-aware feedback and safety-related mechanisms, were designed based on the needs of stakeholders, primarily users and caregivers, throughout the system architecture. ATAM evaluation shows that *IoTMindCare* supports safety and personalization significantly better than current designs. This work provides a flexible, safe, and extensible architectural foundation for IoT-based depression management systems, enabling the construction of optimal solutions that integrate the most effective current research and technology while remaining adaptable to future advancements. *IoTMindCare* provides a unifying, aggregation-style reference architecture that consolidates design principles and operational lessons from multiple prior IoT mental-health solutions, enabling these systems to be instantiated, compared, and extended rather than directly competing with any single implementation.

## 1. Introduction

Depression is a prevalent and growing mental health challenge worldwide, affecting over 280 million people [1]. With increasing modernization, rapid urbanization, and the widespread adoption of digital lifestyles, individuals are experiencing heightened levels of stress, loneliness, and social disconnection, factors that contribute to the onset and exacerbation of depressive disorders [2]. Predictive research studies estimate an increase in the number of people affected by depression by 2030 [3]. This trend highlights the urgent need for innovative, accessible, and scalable approaches to support mental health management, especially within environments where individuals lack regular medical oversight.

Traditional depression management methods rely on episodic assessments and manual reporting, which are often prone to recall bias and delays [4]. In contrast, IoT enables continuous and context-aware monitoring via interconnected devices capable of real-time sensing, transmission, and processing. Wearables, ambient sensors, smartphones, and voice assistants can collectively capture multimodal indicators, heart rate variability, sleep cycles, movement patterns, speech features, and environmental cues to provide a richer, more nuanced monitoring of mental health. This automatic, continuous data stream reduces user burden and has the potential to improve accuracy, enabling a shift from reactive management to proactive and preventive care through immediate anomaly detection and personalized feedback.

Architecturally, most IoT mental health prototypes follow an edge/cloud paradigm [5,6]: sensors transmit data to a local gateway for initial processing, relaying summaries to cloud platforms for deeper analytics and long-term storage. Fog-computing layers are sometimes inserted to reduce latency during emergencies, and standard protocols such as Message Queuing Telemetry Transport (MQTT) or Constrained Application Protocol (CoAP) provide secure, lightweight connectivity between heterogeneous devices. Despite these advantages, no fully commissioned IoT solution for depression management has been deployed at scale. Our structured literature review, presented in Section 2, reveals several persistent barriers, including integration and interoperability issues, privacy and ethical concerns, regulatory and compliance challenges, technological limitations, algorithmic bias, and lack of reference standards.

We hypothesize that a reference architecture that integrates current best practices and prioritizes safety, personalization, and extensibility, can significantly improve the real-world deployment of current and future IoT-based depression management solutions. To test this hypothesis, we follow ADD [7] to develop an integrative reference architecture that integrates the findings from our literature review of current best practice, including emerging standards and domain requirements, and prioritizes safety and personalization. We then use ATAM [8] to evaluate the resulting architecture. Further validation is performed using Unified Modeling Language (UML) models and comparison with benchmark architectures.

The proposed IoT-based reference architecture (*IoTMindCare*) assimilates best practices, emerging standards, and domain requirements into a unified framework shaped around safety and personalization. It features layering in its logical view to support cross-layer coordination, real-time sensing, behavior analysis, and adaptive interventions through user and caregiver feedback. Unlike existing works, our reference architecture is generalizable and standards-informed. It accommodates heterogeneous sensors, applies to diverse usage contexts, and embeds ethical safeguards from the outset. While clinical trials remain future work, we establish a solid foundation for next-generation mental health platforms that are intelligent, adaptive, and ready for real-world deployment. We present *IoTMindCare* as a collection of established architectural patterns and operational insights that make it easier to adapt existing solutions, compare alternatives, and extend them for future needs. Instead of emphasizing one specific anomaly detector, the framework provides a flexible architecture for evaluating and selecting components based on application requirements.

Evaluation and comparison show that *IoTMindCare* is designed to support reductions in FPR and FNR through multi-stage verification, feedback-driven adaptation, and edge-cloud tradeoffs. The quantitative evaluation of these reductions is dependent on concrete instantiations and is left to future prototype and pilot studies.

The main contributions of this research are as follows:

- Identifying core challenges in IoT-based depression management via a structured literature review (Section 2);
- A modular, extensible reference architecture (Section 3) that supports heterogeneous sensors, edge/cloud analytics, safety protocols, and personalization;
- Demonstrating the proposed architecture’s applicability by instantiating a concrete IoT depression care system that delivers proactive, ambient, and user-aware support and related evaluations (Section 4).

**Figure 1:**
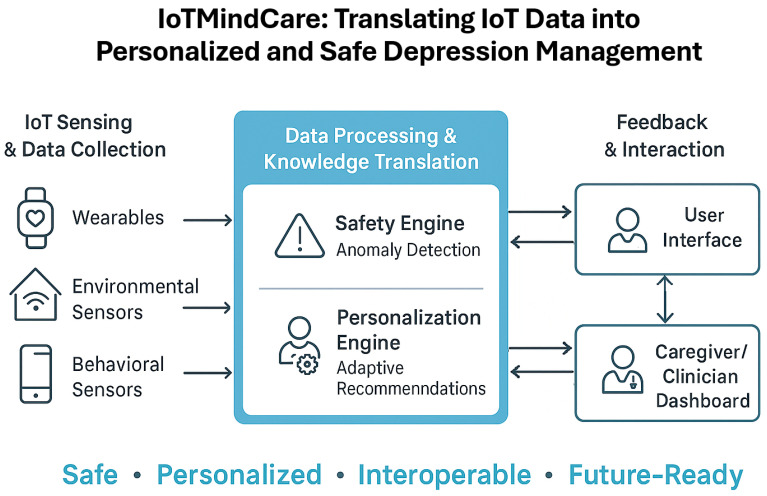
Graphical abstract of the main contributions of *IoTMindCare*.

An overview of the proposed *IoTMindCare* reference architecture illustrating its multi-layered structure is indicated in Figure 1. The diagram highlights the system’s key qualities, including safety, personalization, interoperability, and adaptability.

## 2. Related Works

This review focuses on IoT- and sensor-based solutions that directly inform the design, deployment, and evaluation of reference architectures for continuous, personalized depression management. We surveyed peer-reviewed publications and technical reports from 2016 to 2025 across IEEE Xplore, ACM Digital Library, PubMed, and Google Scholar using keywords including “IoT mental health”, “depression monitoring architecture”, “personalized healthcare IoT” and “ambient assisted living”. We excluded single-purpose apps and non-IoT interventions unless they offered useful contextual integration points. The review prioritizes IoT- and sensor-based solutions that target vulnerable populations, including users at risk of depression and individuals requiring assisted living. This focus ensures that architectural lessons reflect realistic operational constraints and personalization requirements for these user groups.

Prior research clustered into four domains that guided *IoTMindCare*’s architectural drivers and quality-attribute scenarios, as follows:

- **Large-scale smart-home and Activities of Daily Living (ADL)/Human Activity Recognition (HAR) platforms:** Large-scale initiatives and datasets, such as the SMART BEAR consortium [9], which targets heterogeneous clinical and consumer sensors for aging-in-place pilots, and contemporary SPHERE [10] releases with multi-sensor annotated datasets, show how diverse sensor suites can be combined for robust ADL inference and longitudinal baselining. Recent work on virtual smart-home simulators and digital twins provides practical tools for testing ADL recognition and failure modes at scale. Meanwhile, contemporary reviews and studies on HAR system lifespan, semi-supervised learning, and ensemble methods highlight modern approaches for handling concept drift and label scarcity in long-term deployments [11]. These efforts collectively provide updated empirical and methodological evidence that multi-modal fusion, simulator-based validation, and hybrid learning pipelines improve robustness and reduce failure modes. These lessons directly motivated *IoTMindCare*’s modular *Sensing Layer*, fallback sensing strategies, and *Personalization Engine* design.
- **Edge–cloud orchestration and privacy-preserving learning:** Edge–fog–cloud orchestration frameworks and federated/on-device learning solutions, such as FedHome [5], VitalSense [12], and related edge-IoT work, inform placement decisions for training, inference, and data minimization. These works inform our hybrid deployment choices, including edge-first detection for latency and privacy and cloud-based personalization for long-term adaptation, and shaped the *Personalization Engine* and deployment recipes in *IoTMindCare*. Similarly, Benrimoh et al. [13] explore individualized therapy recommendations using deep neural networks. While its focus is on clinical personalization, *IoTMindCare* complements these approaches by providing an architectural foundation to integrate these models within IoT-based monitoring systems.
- **Safety, escalation, and multi-stage verification:** Ambient Assisted Living (AAL) pilots and alarm-management projects, such as [14,15], emphasize the operational need for multi-stage verification and caregiver-in-the-loop escalation to reduce false alarms while ensuring timely response. These operational patterns motivated *IoTMindCare*’s *Safety Engine* and tiered alert/escalation policy.
- **Algorithmic and modality advances (explainability and contactless sensing):** Advances in explainable wearable models and digital-twin/hierarchical detection pipelines provide mechanisms for clinician-facing explainability and hypothesis testing before escalation [6,16]. Complementary research on contactless modalities, such as radar and Wi-Fi CSI, provides privacy-respecting fallback sensing options when wearables are unavailable [17]. *IoTMindCare* integrates these algorithmic and modality-oriented lessons through explainable interfaces and contactless sensing options, with strict local privacy controls in place.

### 2.1. Operational, Privacy, and Standards

Cross-cutting operational lessons include favoring passive, low-burden sensing for adherence; using multi-modal fusion to reduce false positives; limiting raw-data transmission via on-device preprocessing; and integrating caregiver consent and verification flows.

Standards and middleware, such as HL7 FHIR [18], OpenEHR [19], FIWARE [20], and IEEE 11073 [21], provide concrete data models and connectors that make interoperability and safe clinical integration feasible. *IoTMindCare* adopts these as normative recommendations for semantic integration and secure exchange.

### 2.2. Synthesis: How the Literature Shaped IoTMindCare

The surveyed works are operational inputs rather than competing end-products. They shaped *IoTMindCare* as follows:

- **Sensing layer:** multi-modal fusion, fallback sensing, and passive modalities from ADL/HAR platforms inform sensor selection and local rules.
- **Safety Engine:** multi-stage verification and caregiver-in-the-loop designs from AAL pilots shape alert policies and escalation workflows.
- **Personalization Engine:** federated and edge–cloud learning patterns guide where to place model updates and how to preserve privacy while enabling per-user adaptation.
- **Deployment and standards:** edge–fog–cloud orchestration patterns and interoperability standards guide practical integration and long-term maintainability.

For context, we retain listing therapeutic and self-report systems, such as Woebot [22], Wysa [23], BetterHelp [24], Moodfit [25], and T2 Mood Tracker [26], as possible integration endpoints or manual-input channels.

Table 1 summarizes representative IoT, ADL/HAR, and edge-cloud projects and research that directly inform *IoTMindCare* across sensors, personalization, safety, and deployment patterns.

In summary, the literature provides both Technical Building Blocks, including sensing, edge–cloud orchestration, safety, and personalization, and Technical Constraints, including limited resources, privacy, and device heterogeneity, that collectively informed the architectural drivers, Quality Attribute Scenario (QAS), and component-level decisions in *IoTMindCare*.

## 3. IoTMindCare: Proposed Reference Architecture

*IoTMindCare* is built using ADD where we first identify key architectural drivers (Section 3.1) to the develop architectural views (Section 3.2).

### 3.1. Architectural Drivers

#### 3.1.1. Functional Requirements

Our literature review points to five core functional requirements:

F1 **Continuous Monitoring** or sensing of physiological, behavioral, and environmental signals.
F2 **Context-Aware Decision-Making** of multimodal data within spatial-temporal and behavioral contexts.
F3 **Alert Generation and Escalation** to caregivers or clinicians in response to detected risk conditions.
F4 **User and Clinician Interfaces** for stakeholders.
F5 **Personalized Feedback** and intervention mechanisms tailored to individual users.

#### 3.1.2. Quality Attribute Scenarios

Safety and personalization are the key quality attributes in our architecture design process, and are captured using the quality-attribute scenario template [27].

**QAS1: Safety:** The system must detect critical events, such as depressive crises or self-harm, and respond with timely alerts using a probabilistic, context-aware anomaly detection model (Table 2). Detection accuracy, timeliness, and escalation logic are critical. This includes handling uncertainty through a confidence scoring model and minimizing the False Positive Rate (FPR) and False Negative Rate (FNR) as primary operational objectives. Events are cross-referenced with historical and contextual data to improve detection reliability.
**QAS2: Personalization:** The system must adapt dynamically to users’ physiological and behavioral baselines, with support for partial or heterogeneous device configurations (Table 3). When sensors are missing, alternative data (manual input, AI-inferred metrics) must substitute for automated sensing. Personalization includes dynamic switching between modes, user-specific thresholds, and real-time updates to decision logic.

Recent IoT-health and applied risk-detection studies explicitly report false positive and false negative outcomes when evaluating anomaly and crisis detection models. For example, Gupta et al. [6] show how hierarchical/federated architectures reduce false positives via multi-layer model aggregation and context validation, motivating multi-stage verification in edge/cloud pipelines. Similarly, applied suicide-risk prediction studies report sensitivity/specificity and precision metrics to quantify clinical detection trade-offs and operational risk [28]. These studies motivate our choice to evaluate safety and personalization using FPR and FNR alongside latency and robustness measures in our reference architecture.

To support reproducible validation of the QASs, we specify measurement protocols and metrics that should be applied when an implementation or simulation of IoTMindCare is constructed. For QAS1 (Safety), we recommend reporting FPR and FNR, sensitivity (recall), specificity, detection latency (time from event onset to alert), and escalation latency (time from alert to caregiver/clinician notification). For QAS2 (Personalization), we recommend adaptability, installability, customization, co-existence adequacy, and reliability [29]. Validation should use temporally aware splits to reflect longitudinal deployment and expose concept drift. Calibration metrics (e.g., Brier score or reliability diagrams) are recommended when probabilistic confidence scores drive escalation thresholds.

To support reproducible validation of the QASs, we specify measurement protocols and metrics that should be applied when an implementation or simulation of IoTMindCare is constructed. For QAS1 (Safety), we recommend detection metrics such as True Positive Rate/Sensitivity, FPR, FNR, Specificity, and Precision, together with operational metrics, including detection latency (time from event onset to alert) and escalation latency (time from alert to caregiver/clinician notification). For QAS2 (Personalization), we recommend measures of personalization gain, such as absolute and relative reductions in FN and FP rates after per-user adaptation compared to a population model (such as FNR and FPR), model convergence speed (time or number of examples to reach stable per-user performance), and robustness under sensor loss (percent performance drop when a subset of sensors is removed). Validation should use temporally aware splits (training on earlier windows, testing on later windows) to reflect longitudinal deployment conditions and to expose concept drift. Finally, calibration metrics (such as Brier score or reliability diagrams) should be used where probabilistic confidence scores drive escalation thresholds.

#### 3.1.3. Technical Constraints

The key technical constraints highlighted by our review [5,6,30] are as follows:

TC1 **Limited Edge Resources** necessitate light-weight and distributed processing.
TC2 **Privacy Constraints** require local processing and adherence to privacy legislation.
TC3 **Device Heterogeneity** points to the need to tolerate and adapt to variable device configurations.

### 3.2. Architecture Views

The architecture is presented using the 4+1 view model [31]. Each view reports a different but equally critical aspect of the architecture and is linked to a different combination of architectural drivers.

#### 3.2.1. Logical View

Logically, the architecture has a layered structure due to this pattern’s robustness in managing modularity and ensuring logical separation between sensing, processing, and interface components (Table 4). Layering is widely adopted in existing solutions [32,33]. Other architectural styles, such as microkernel and service-oriented architecture, are less suitable due to weaker support for reactive and real-time event handling required by QAS1, and unacceptable latency for context-sensitive, time-critical operations, respectively.

This view enhances the structure from [34] by embedding user-specific personalization across all layers and enabling dynamic thresholding, offering resilience (TC3) and deeper alignment with QAS2. This enhancement improves system adaptability and responsiveness beyond prior work.

Each layer supports specific architectural drivers. The Sensing layer provides real-time signal capture support (F1, QAS1). The Network and Communication layer ensures reliable data routing (F2, TC3). Data Processing and Storage enables edge/cloud processing (F2, QAS1, QAS2). The Service layer implements core decision logic (F2, F3, QAS1). The Interface layer provides personalized feedback and real-time alerting (F3–F5, QAS2).

#### 3.2.2. Development View

**Figure 2:**
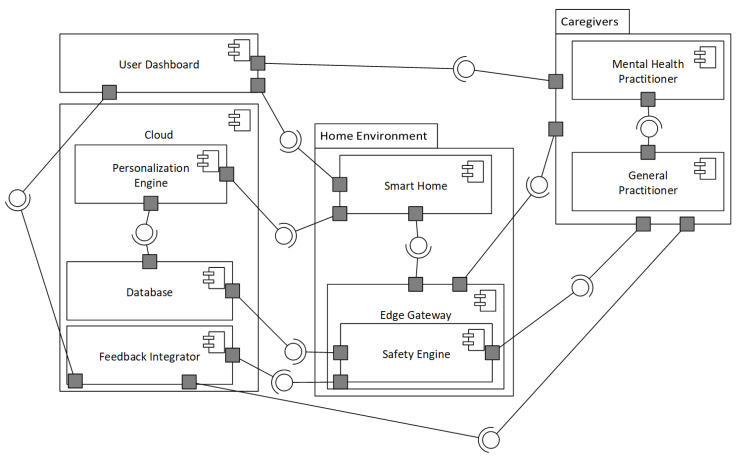
Development view—UML Component diagram showing the major software modules for smart home, cloud, and user environments.

The development view (Figure 2 defines the internal structure and interactions between software modules distributed across smart home environments, cloud infrastructure, and external actors such as users and caregivers.

The development view adopts a component-based architecture with RESTful and message-passing interfaces due to support of loose coupling, deployment flexibility, and fault isolation [35,36,37]. This pattern enables modular, distributed services that feature real-time responsiveness and adaptability.

Unlike fixed-rule systems like CarePredict [38] using predefined activity models, *IoTMindCare* dynamically adapts to each user’s data through the cloud-based Personalization Engine. Feedback loops are integrated via the Feedback Integrator, improving the system over time. This enhancement is central to achieving QAS2 under real-world device constraints (TC3), highlighting adaptability and scalability.

Smart Home edge interface performs continuous monitoring (F1) and forwards data to the Edge Gateway, which executes lightweight inference and handles fallback logic during cloud disconnection (QAS1, TC1, TC2). The Safety Engine performs anomaly detection using probabilistic models and generates alerts based on severity thresholds (F3, QAS1). The cloud-based Personalization Engine manages user-specific models and dynamically adjusts service logic based on behavioral patterns (F5, QAS2, TC3). Moreover, the Feedback Integrator incorporates feedback from users and caregivers to refine system behavior (F5, QAS2), while the Central Database stores longitudinal data and personalization parameters.

For user interaction, Mobile Application enables real-time feedback, notifications, and manual data entry for user reflection, alert acknowledgment, and configuration (F4, F5). Clinician/Caregiver Dashboard provides real-time and retrospective views of user states, anomaly logs, and behavioral trends for escalation actions and threshold customization (F3, F4, QAS1).

#### 3.2.3. Process View

**Figure 3:**
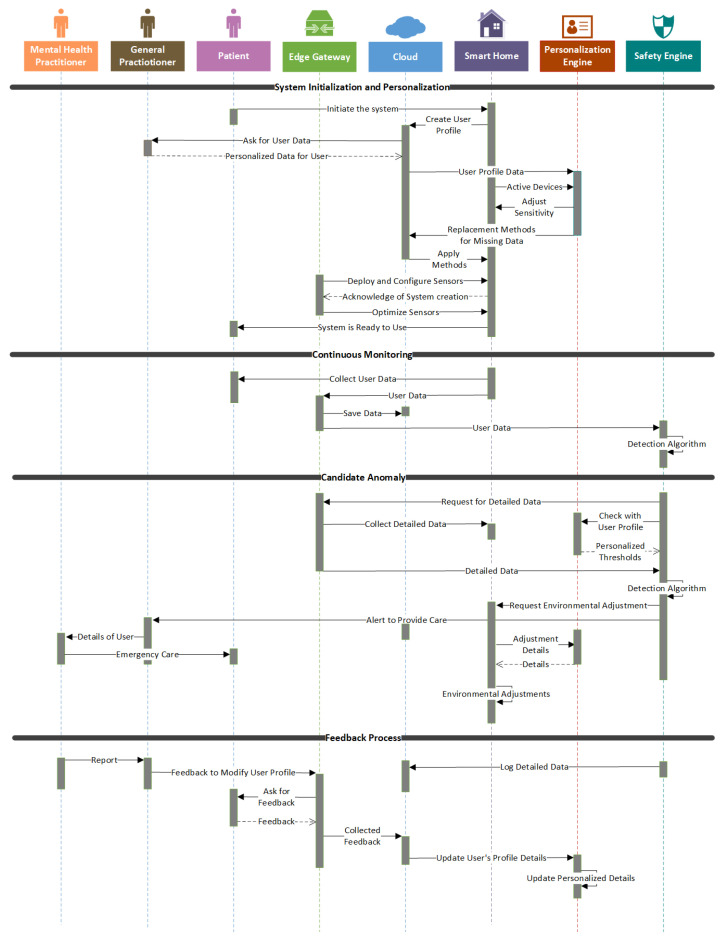
Process view—UML Sequence Diagram showing end-to-end system behavior categorized into four stages and featuring dynamic interactions among smart home, cloud, and user interfaces.

The process view (Figure 3) describes the system’s dynamic behavior, capturing the interaction patterns among core components during real-time operation. It adopts an event-driven architectural style, well-suited for reactive, asynchronous IoT-based health monitoring systems due to its responsiveness and scalability [39,40]. It also uses a client–server pattern, where cloud services hold stateful logic, while edge and user devices act as clients, emitting or receiving events.

Unlike rule-based detection chains in [34], this view incorporates closed-loop adaptation, probabilistic scoring, and scalable escalation, offering resilience and evolving personalization aligned with real-world deployment challenges.

We propose four key processes reflecting the system’s end-to-end lifecycle: **System Initialization and Personalization, Continuous Monitoring, Candidate Anomaly Detection**, and **Feedback Integration**. System Initialization and Personalization begins during setup or after reconfiguration. General Practitioner provides user-specific information such as baseline activity and risk thresholds. The cloud-based Personalization Engine generates contextual thresholds and fallback logic for sensor gaps (F5, QAS2, TC3).

In Continuous Monitoring, Edge Gateway gathers and preprocesses multimodal data. Obvious anomalies are flagged locally, while filtered data is sent to the cloud for deeper analysis. Real-time and historical data are stored centrally (F1, F2, TC1, TC2). In Candidate Anomaly Detection and Alert Escalation, Safety Engine evaluates deviations using probabilistic models. If an anomaly is detected, multi-level alerts are triggered: from users to general and mental health practitioners, depending on severity (F2, F3, QAS1). Finally, in Feedback Integration and Adaptation, users and clinicians provide feedback through dashboards. Feedback Integrator refines the personalization logic and detection thresholds, enabling continuous learning (F5, QAS2, TC3).

#### 3.2.4. Deployment View

**Figure 4:**
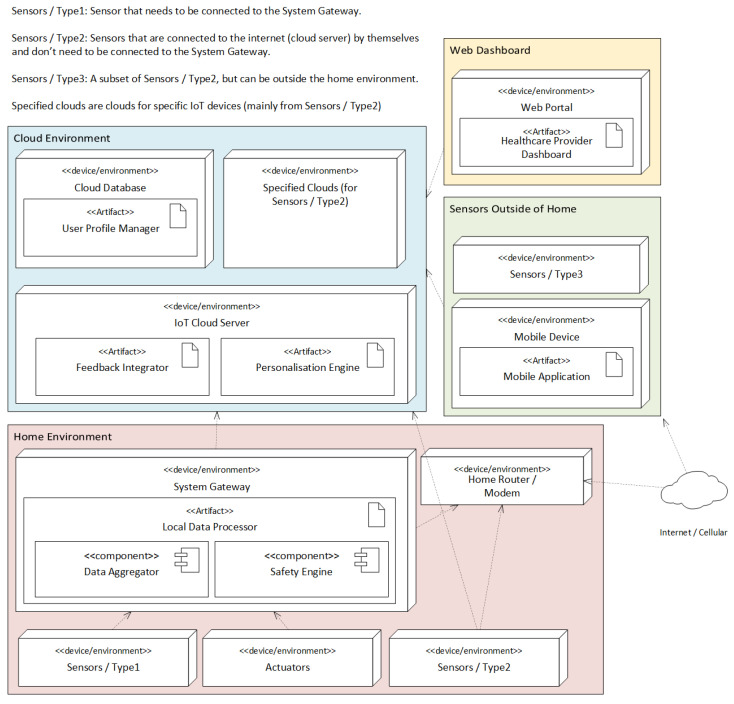
Deployment view—UML Deployment diagram showing the mapping of the development view components to physical or infrastructure resources.

The deployment view illustrates how software components are physically distributed across edge devices, cloud infrastructure, and stakeholder interfaces (Figure 4). This view relates to latency, fault tolerance, and scalability requirements. A hybrid edge–cloud deployment strategy is adopted, a common best practice in current works [41,42].

The deployment view enables scalability and resilience by decoupling edge and cloud operations. Cloud components can be updated dynamically without disrupting critical edge functions. Communication protocols are optimized for IoT reliability and low-latency demands, aligning with system constraints (TC1–TC3) and quality attributes (QAS1, QAS2).

Multiple Sensing Devices capture multimodal behavioral and physiological data in real time. These feed into a local Smart Home Gateway, which hosts the Edge Gateway and Safety Engine. This setup supports on-site anomaly detection, buffering, and fallback logic during cloud disconnections (QAS1, TC1).

The Cloud Infrastructure manages the Personalization Engine, Feedback Integrator, and long-term Database. It handles high-complexity tasks such as behavioral analytics, adaptive model updates, and secure storage. Encrypted communication between the cloud and smart home ensures privacy and integrity (QAS2).

Users and caregivers access the system through Mobile Applications and Clinician Dashboards, which interface primarily with cloud services for alerts, feedback, and system configuration. These apps can also connect locally for real-time updates when needed.

## 4. Architecture Evaluation

We evaluate the proposed *IoTMindCare* using ATAM [8,43] for its support for the primary QAs, safety and personalization, as well as complementary 4+1 UML views (See Section 3). The QA scenarios from Section 3.1.2 were analyzed to ensure that they are holistically addressed by the architecture, across all relevant views. Table 5 details how each QA is supported by *IoTMindCare*, and indicates to a robust architecture delivering safe and personalized IoT-based depression management.

Tradeoff Analysis

The architectural choices must also balance the tradeoffs between *safety* and *personalization*, particularly in minimizing the FPR and FNR in behavioral anomaly detection. We identified the following critical tradeoffs:

1. **Safety vs. Personalisation:** A central tradeoff exists between safety and personalization. Personalization improves detection sensitivity (reducing FNR) by adapting to individual baselines; however, overly narrow per-user thresholds may increase FPR if not validated. IoTMindCare mitigates this via a tiered verification pipeline (edge trigger → cloud contextual validation → caregiver confirmation) and feedback-driven recalibration. However, overly personalized thresholds risk increasing FPs if not rigorously validated. This tradeoff is mitigated by delegating final anomaly verification to a centralized cloud component (Figure 2) and integrating feedback from caregivers (Figure 3) to calibrate and improve models iteratively.
2. **Feedback Loops vs. Timeliness:** Incorporating caregiver and user feedback into anomaly detection loops (Figure 3) helps reduce FPR/FNR rates over time. However, this introduces delays in model retraining and potential inconsistencies during transition phases. The architecture mitigates this by decoupling feedback ingestion from real-time alerting, maintaining responsiveness while still enabling long-term model refinement.
3. **Local Autonomy vs. Central Oversight:** To ensure availability and responsiveness, the system gateway performs initial anomaly evaluation at the edge (Figure 4). This supports low-latency decision-making and offers resilience during cloud disconnection, contributing to safety (FNR reduction). However, relying solely on edge intelligence limits access to broader contextual information needed to reduce FPRs. As a result, the architecture introduces a tiered decision-making pipeline: edge components perform preliminary assessments, while cloud services provide contextual validation to reduce FPRs. This design reflects a deliberate trade-off between local autonomy and central oversight.

*IoTMindCare* intentionally aggregates capabilities from the surveyed literature so that many existing architectures can be expressed as subsets or specific instantiations of the reference model. This framing avoids claiming direct superiority; instead, the contribution is a unifying architecture that (a) makes feature/metric tradeoffs explicit, (b) clarifies where variability points should be placed to compare detectors (e.g., choice of anomaly model, thresholding strategy, retraining cadence), and (c) provides concrete recipes for extending prior designs toward more robust, future-proof deployments.

Overall, *IoTMindCare* carefully balances quality attributes, with safety and personalization as primary drivers. Modularity, layered processing, and feedback integration strategies explicitly addressed tradeoffs, as evidenced in the UML diagrams and quality scenario mappings.

### 4.1. Comparison with Existing Architectures

Table 6 provides a high-level comparison between IoTMindCare and selected contemporary IoT-health frameworks across data sources, personalization, edge/cloud strategy, explainability, and multi-stage anomaly handling.

This comparison focuses on architectural patterns, operational principles, and design choices rather than empirical performance metrics, as IoTMindCare is a reference architecture.

Our architecture combines explainability and personalization within a hierarchical edge-cloud structure, while having other levels of safety and personalization. It extends these through the following:

1. Incorporating home environmental sensors.
2. Employing multi-stage anomaly verification to manage FPR/FNR tradeoffs.
3. Supporting feedback-driven model adaptation with user/caregiver input.
4. Enabling edge-first inference policies for low latency and privacy.

While Zhang et al.’s model [16] presents strong anomaly detection within a controlled dataset, it has a wearable-only scope and centralized deployment, which limits generalization and real-world responsiveness. In contrast, our system is designed for continuous deployment, with added robustness mechanisms (like model updates from feedback) and explainable alerts through transparent processing stages. Compared to federated learning systems, our architecture relies on explicit feedback loops rather than fully distributed updates, offering a more straightforward path to per-user tuning and safety in mental health contexts.

These comparisons suggest that our architecture is a more holistic solution for real-world, mental health IoT deployment, better suited to privacy, safety, personalization, and real-time operation.

### 4.2. Scenario-Driven Instantiation and Configuration

To demonstrate the applicability of *IoTMindCare* in realistic deployments while preserving its role as a high-level reference architecture, we provide a set of concrete IoT-driven scenarios drawn from the literature, along with a mapping of these scenarios to the architecture. For each scenario, we indicate relevant literature sources, the architecture views most involved, the primary QAS affected, and brief configuration guidance. These examples are meant as practical guides for building, testing, or running pilot versions of systems.

#### Representative Scenarios

1. **Early deterioration detection (gradual mood decline):** Long-term ADL/sleep/activity drift that signals worsening depression (motivated by SMART BEAR [9]).
2. **Acute crisis detection (self-harm/suicidal risk):** Rapid onset of high-risk states requiring low-latency detection and escalation (inspired by AAL pilots and safety studies [14,15]).
3. **Sleep disturbance and insomnia monitoring:** Night-time patterns detected via bed/pressure and respiration (contactless modalities), indicating sleep disruption associated with mood change (motivated by SPHERE, contactless sensing work [10,17]).
4. **Social isolation/activity withdrawal:** Reduced outings, fewer device interactions, reduced social communications extracted from ADL/HAR traces (CASAS [44] and SPHERE [10]).
5. **Medication non-adherence/routine deviation:** Missed medication or routine changes inferred from smart-plug, cabinet-door, and activity sensors (SMART BEAR [9] AAL studies [14,15]).
6. **Acute stress episode (physiological spike):** Short-term HRV and activity spikes detected by wearables requiring brief interventions or prompting (wearable explainability studies [16]).
7. **Contactless monitoring for privacy-sensitive users:** Use radar/Wi-Fi channel state information and on-device inference where wearables are infeasible (contactless systems [17]).
8. **Teletherapy-triggered sensing:** Trigger richer sensing or clinician contact when conversational agents or self-report apps indicate increased risk (integration with digital therapeutics).

For a given deployment, practitioners can use the recipes above to configure IoTMindCare as follows:

- **Select sensors and signals** relevant to the scenario
- **Choose model placement:** edge-first for low-latency safety scenarios; cloud-enabled personalization for long-term baselining and retraining.
- **Define escalation policy:** map detection confidence levels to tiered actions (edge prompt, cloud validation, caregiver/clinician alert) within the Safety Engine.
- **Personalize and test:** initialize thresholds from population priors, then adapt per-user via the Feedback Integrator. Use concrete quantitative validation metrics, such as reductions in FNR, reductions in FPR, improvements in sensitivity/specificity, and changes in detection and escalation latency, to evaluate the personalization gain.
- **Privacy and failover:** apply on-device preprocessing, encrypted model updates, or federated learning, and contactless fallbacks where wearables are refused.

These scenario instantiations are explicitly intended to guide future prototype or simulation studies. For each scenario, we recommend a pilot that measures the QA metrics listed above (FPR/FNR, detection latency, and personalization gain), with clearly defined A/B comparisons or pre- and post-personalization analyses. These evaluation plans are consistent with the ATAM-driven analysis and the QA metric framework introduced in Section 3.1.2.

**Empirical evaluation plan (guidelines).** Because IoTMindCare is a reference architecture, empirical claims about reductions in FPR/FNR require the instantiation of prototypes and controlled evaluation. For future prototypes we recommend the following evaluation regimen: (1) choose scenario(s) from Table 7; (2) run pre/post personalization experiments where the baseline uses population thresholds and the experimental arm uses IoTMindCare personalization and feedback loops; (3) report FPR, FNR, sensitivity, specificity, detection latency, escalation latency, and calibration (Brier score); (4) include robustness tests: sensor dropout (remove sensor modalities), concept-drift injection (temporal distribution shifts), and realistic network outages; (5) present results as absolute and relative changes in FPR/FNR or bootstrap estimates to quantify uncertainty.

### 4.3. Limitations and Threats to Validity

We summarize key limitations of *IoTMindCare* as follows, adapted from standard research design frameworks such as the SPHERE deployment framework [10]. These include threats to validity in system evaluation, deployment feasibility, and ethical compliance. While our work lays a conceptual and architectural foundation, future empirical studies are required to address these threats.

- **Construct Validity:** Behavioral and physiological signals may not fully capture the complex psychological aspects of depression and have not been clinically validated against gold-standard diagnostic tools.
- **External Validity:** The system has not yet been deployed across diverse demographics, socioeconomic groups, or cultural contexts, limiting generalizability.
- **Ethical and Legal Constraints:** The system has not undergone formal ethical review or compliance assessment with regulations such as GDPR or HIPAA, especially regarding passive data collection.
- **Technical and Ecological Validity:** Assumes consistent sensor reliability, user compliance, and stable network conditions; real-world variability and behavioral shifts due to monitoring awareness may affect performance.

The validity issues include methodological concerns, such as the lack of clinical validation and the absence of empirical evaluation, as well as practical limitations in real-world deployment and ethical compliance. Specific challenges include construct and internal validity gaps, potential algorithmic bias, and uncertainties in scalability under resource constraints. Although the architecture presents a promising direction for intelligent depression care, future work must empirically validate its components, address ethical safeguards, and ensure robustness across user populations.

## 5. Conclusions

This research proposes a modular, layered IoT-based reference architecture (*IoTMindCare*) for continuous and personalized depression care in non-clinical settings. Integrating multi-modal sensing, edge-cloud analytics, adaptive feedback loops, and safety-driven design principles, our architecture addresses key challenges identified in existing literature, particularly safety, personalization, real-time response, and architectural extensibility. Through comparative analysis and design modelling, we demonstrated how *IoTMindCare* offers a comprehensive framework to guide the development of intelligent mental health monitoring systems.

While *IoTMindCare* offers a strong conceptual and technical foundation, several opportunities remain for future advancement. Real-world deployment and clinical trials would be instrumental in validating the system’s effectiveness in diverse populations. Adaptive learning components may be incorporated to support long-term personalization and reduce false positive or negative detections. Additionally, integration with healthcare infrastructures such as electronic health records and alignment with privacy and regulatory standards could support broader adoption. Moreover, the prototype can be strengthened by simulating a real-world environment. These directions represent promising avenues for translating IoT-based mental health architectures from prototype to practice.

## Figures and Tables

**Table 1:** Comparison of recent IoT-based mental and physical health monitoring architectures, their characteristics, and associated benefits.

System/Reference	Focus Area	Key Technologies	Unique Contributions/Features	Limitations Addressed by IoTMindCare
SMART BEAR [9]	Healthy aging, large-scale IoT monitoring	Heterogeneous sensors, AI-based analytics, cloud integration	Personalized interventions for older adults using real-world evidence; scalable data fusion platform	Focuses on physical health; lacks adaptive stress and mood analytics
SPHERE [10]	Multi-sensor home monitoring, ADL/HAR	Video, wearable, and environmental sensing; ML-based ADL inference	Provides open datasets and multimodal monitoring for chronic conditions	Does not address personalization or dynamic emotional context
CASAS [11]	HAR and anomaly detection	Semi-supervised ensemble learning, temporal features	Enhanced HAR accuracy in complex environments	No user-centered personalization or mental health context
VitalSense [12]	Smart city and remote health monitoring	Edge–fog–cloud hierarchy, digital twins, adaptive routing	Low-latency IoT architecture for continuous health monitoring	Lacks domain-specific decision logic for emotional wellbeing
AAL Healthcare 5.0 [14]	Assisted living, healthcare personalization	IoT–AI integration, context awareness, user modeling	Personalized ambient intelligence and health prediction	No explainable AI or caregiver feedback integration
AAL AI Review [15]	AI in AAL	ML and DL algorithms, cognitive computing, privacy concerns	Comprehensive review highlighting challenges in transparency and interoperability	IoTMindCare addresses these gaps via explainable reasoning and standardized APIs
Explainable Wearable Monitoring [16]	Depression/anxiety detection from wearables	Explainable anomaly detection, multimodal fusion	Transparent AI model linking sensor data to mental state changes	Lacks a modular IoT reference architecture or cross-platform interoperability
Contactless IoT Health [17]	Vital signs and ADL tracking	Radar, thermal, and computer vision-based sensing	Comprehensive non-invasive monitoring system with motion tracking	No personalized feedback or multi-stakeholder (user–clinician) data flow
Digital Therapeutic Apps [22,23,24,25,26]	AI chatbots, cognitive behavioral therapy, tele-therapy	Natural Language Processing-based conversational agents, self-tracking apps	Real-time emotional support and mood tracking through mobile interfaces	Standalone, not integrated with physiological or contextual IoT data

**Table 2:** Scenario for requirement safety.

**Stimulus**	The system detects unusual patterns (such as unusual inactivity or negative self-talk) in the incoming data streams.
**Stimulus Source**	The user The system itself Sensors/devices
**Environment**	The system is running under normal operation.
**Artifact**	Depression management system components: sensing modules, monitoring modules, communication interfaces, caregiver dashboard, and alert/logging mechanisms.
**Response**	The system determines whether observed patterns meet the criteria for a critical or high-risk event and decides whether escalation is required. If escalation is needed, the system triggers local and remote alerts and records the event for auditing and traceability.
**Response Measure**	**Primary objective:** minimize FN (missed legitimate emergencies), aim as close to zero as practicable for the scenario. Studies emphasize maximizing detection sensitivity to avoid missed emergencies, even when this increases false alarms. Zhang et al. [16] reported 12% FN (recall = 0.88), while Wu et al. [5] achieved 95% overall accuracy without explicit FP/FN reporting, providing indicative performance ranges. **Secondary objective:** minimize FP subject to the primary objective. When FN performance is equal, prefer the lower FP option. In Zhang et al. [16], the corresponding precision is 0.73 implies roughly 27% FP, illustrating the typical trade-off between high sensitivity and increased false alarms. As noted by Gupta et al. [6], multi-stage validation and cross-modal analysis can effectively reduce such false alarms while preserving safety sensitivity. **Timeliness:** Ensure prompt detection and escalation in a timely manner related to the event. Acceptable latency depends on context, consistent with timelines reported in the SMART BEAR [9] and SPHERE [10] frameworks. **Auditability:** 100% of the alerts should include contextual and explainable data for human review and post-event analysis, aligning with safety validation requirements observed in prior IoT-based behavioral monitoring systems (Gupta et al. [6] and Zhang et al. [16]).

**Table 3:** Scenario for requirement personalization.

**Stimulus**	The system needs to tailor its monitoring and intervention strategies to the individual user’s characteristics and context (e.g., a user with a specific set of devices and initial profile/context, registers and begins using the system, or an existing user’s context changes, new devices, changed environment, or changed clinical needs).
**Stimulus Source**	The user (profile, preferences, clinical notes, historical patterns) The device inventory and environment (available sensors, connectivity) System-detected context changes (new data streams or changed behavior)
**Environment**	Normal operation with diverse user devices, variable connectivity and technology availability, and differing caregiver support models; personalization must operate under these varying constraints.
**Artifact**	Personalization Engine and related components in the depression-management system (user profile module, feedback integrator, device-adapter/fallback logic, personalization policy store, and caregiver/clinician interfaces).
**Response**	The system can be configured according to the user’s identity, context, and available technology, considering who the person is, their mental health needs, and their environmental setup. The system has high interoperability, adapting to sensor diversity, where multi-device integration supports continuous monitoring across heterogeneous environments. When specific data streams are missing, the system applies fallback or replacement strategies, consistent with the modular adaptation approaches.
**Response Measure**	**Adaptability:** proportion of functions operating under changed conditions (e.g., SMART BEAR adapts to heterogeneous sensors [9]). **Installability/Customization Rate:** time per configuration/number of supported configurations (e.g., SPHERE supports context-dependent deployments [10]). **Interoperability Support Rate:** ratio of supported device/format pairs to total expected (e.g., Wu et al. integrate multiple smart home devices [5]). **Co-existence Adequacy:** ability to work alongside existing systems/devices (e.g., Gupta et al. integrate with existing IoT platforms [6]). **Replaceability Rate:** percentage of modules swappable without affecting operations (e.g., Zhang et al. propose modular anomaly detection [16]).

**Table 4:** Logical view—layering diagram showing the five architectural layers of the architecture and their mapping to safety and personalization.

**Interface Layer** (user interface on mobile devices and actuators, caregiver’s access via dashboard, and feedback mechanism to upgrade the system)	**Personalization** (supporting device variability, customized treatments, user-specific thresholds, and behavioral diversity)	**Safety** (anomalous behavior detection by continous monitoring, real-time alert to caregivers, and environemtal adjustments)
**Service Layer** (real-time monitoring, automated alerts by decision-making, environmental adjustments)		
**Data Processing and Storage** (local processing for real-time and rule-based triggers, cloud processing for long-term behavioral analytics, and data storage)		
**Network and Communication** (local communication, gateway, remote communication, ensuring reliable data exchange between system components		
**Sensing** (collect multimodal data from physiological, environmental, and behavioral sensors, depending on sensors’ availability)		

**Table 5:** Mapping Quality attribute scenarios to architectural evidence.

QAS	Relevant Components/Diagrams	Validation Evidence	Component Level Mapping (Figure 2)	Behavioral-Level Mapping (Figure 3)	Deployment-Level Mapping (Figure 4)
Safety (FPR) (Table 2)	Safety Engine (Figure 2 and Figure 4)—Alert Generation (Candidate Anomaly Section in Figure 3))	Alert verification by checking with the edge gateway to reduce incorrect alerts	Multi-layer verification across the System Gateway and Cloud	Reduced by context-aware confirmation in the cloud before triggering alerts	Ensuring local anomaly detection continues during network downtime (edge computing on the System Gateway)
Safety (FNR) (Table 2)	Safety Engine (Figure 2 and Figure 4)—Caregiver and user feedback by Feedback Integrator (Feedback Process Section in Figure 3)	Feedback integration to detect missed anomalies	Caregivers and User interface to facilitates feedback loops and missed-event reporting	Addressed via caregiver and user feedback paths feeding into the anomaly model	Ensuring local anomaly detection continues during network downtime (edge computing on the System Gateway)
Personalization (Figure Table 3)	Personalisation Engine (Figure 2 and Figure 4)—User Dashboard (Figure 2 and Figure 4)—User Profile on Cloud (Figure 4)—Integrating different sensors (Figure 4)—Ask user data from General Practitioner (Figure 3)—Adjust devices and sensitivity (Figure 3)	Adaptable thresholds—Behavioral baselining in algorithms—User-specific anomaly models by checking with user profile—Setting up based on the user’s devices	User module to build user-specific behavioral models and adapt thresholds over time—System configuration based on the user’s specified devices	Individualized thresholds used during verification, and using the user’s available set of devices	Hosting behavioral models and user data in the cloud enables modifications as user thresholds change

**Table 6:** Comparison with recent IoT-health architectures.

Feature	Zhang et al. [16]	Wu et al. [5]	Gupta et al. [6]	IoTMindCare
Data Sources (See Logical View in Section 3.2.1)	Wearables	Wearables/Home Sensors	Wearables/Digital Twins	Wearables/IoT/Environment
Personalization (See Process View in Section 3.2.3)	Global Model	User-Specific Models	Group-Based Models	User Profile/Feedback-Driven
Explainability (See Development View in Section 3.2.2)	SHAP	–	–	Human-in-Loop/Logic Layers and Modules
FPR/FNR Strategy (See Process View in Section 3.2.3	Global Threshold	Personalized FL	Multilevel Validation	Multi-Stage/Feedback
Deployment Topology (See Deployment View in Section 3.2.4)	-	Edge-Cloud Based FL	Hierarchical Edge–Cloud	Hybrid Edge–Cloud with Fallback and Local Safety Logic

**Table 7:** Scenario to architecture mapping: signals, literature sources, relevant views, primary QAS and practical configuration notes for prototype instantiation.

Scenario	Key Signals/Sensors	Source (Examples)	Relevant Views	Primary QAS & Config Notes
Early deterioration detection	Activity patterns, sleep trends, phone/PC usage, longitudinal HR/ actigraphy	SMART BEAR [9]	Logical, Deployment, Process	**QAS2.** Longitudinal baselining in Personalization Engine; cloud-hosted model training with scheduled recalibration; edge summarization to preserve privacy. Use drift-detection and periodic retraining hooks; measure FPR, FNR, sensitivity, specificity, detection latency, and convergence rate.
Acute crisis detection	HRV, speech content/prosody, sudden inactivity, environmental context	AAL reviews/safety pilots [14,15]	Service, Process, Deployment	**QAS1.** Edge-first low-latency detection (Safety Engine) with multi-stage confirmation: edge trigger → cloud contextual validation → caregiver escalation. Configure conservative thresholds + high-confidence escalation; log detection latency and sensitivity/specificity.
Sleep disturbance monitoring	Bed-pressure, respiration (radar/CSI), actigraphy	SPHERE [10], Li et al. [17]	Logical, Deployment	**QAS2** and **QAS1.** Use contactless modalities where wearables unavailable; process on edge for privacy; personalize sleep thresholds per-user; validate with sleep-specific sensitivity score and detection-latency metrics.
Social isolation/ withdrawal	Reduced movement, decreased device interactions, fewer outgoing calls	CASAS/HAR literature [10,11]	Logical, Process, Development	**QAS2.** Personalization Engine tracks social-activity baselines; thresholds adapted via Feedback Integrator; A/B style trials to compare static vs adaptive thresholds; measure reduction in FNR for isolation detection.
Medication/routine deviation	Smart-plug, cabinet/door contacts, schedule adherence logs	SMART BEAR [9], AAL reviews [15]	Development, Deployment	**QAS1 & QAS2.** Edge acknowledgement prompts (in-situ) before escalation; local rule filters + cloud validation; integrate clinician-configured escalation policies. Monitor FPR rate of missed-medication alerts.
Acute stress episode	Wearable HR/HRV, accelerometer, short-term speech features	Explainable wearable studies [16]	Logical, Service	**QAS1 & QAS2.** Edge/local detection for low latency, short-term buffering to cloud for contextual validation; use explainability output for clinician dashboards; evaluate with precision/recall and detection latency.
Contactless privacy-sensitive monitoring	Radar, Wi-Fi CSI, on-device feature extraction	Li et al. [17], contactless sensing literature	Logical, Deployment	**QAS2** and privacy constraints. Prefer on-device feature extraction and encrypted summaries; disable raw audio/video transmission; define privacy-preserving model update policies (differentially private updates or federated patterns).
Teletherapy- triggered sensing	Self-report, chatbot flags, scheduled clinician prompts	Digital therapeutics/ chatbots [22,23]	Interface, Process, Development	**QAS2.** Use chatbot signals to change sensing mode (e.g., increase sampling), start short-term local analytics, and route data to clinician dashboards; measure user acceptability and engagement.

## Data Availability

The original contributions presented in this study are included in the article. Further inquiries can be directed to the corresponding author.

## Abbreviations

The following abbreviations are used in this manuscript:

IoT	Internet of Things
ADD	Attribute-Driven Design
ATAM	Architecture Trade-off Analysis Method
MQTT	Message Queuing Telemetry Transport
CoAP	Constrained Application Protocol
UML	Unified Modeling Language
ADL	Activities of Daily Living
HAR	Human Activity Recognition
AAL	Ambient Assisted Living
QAS	Quality Attribute Scenario
FPR	False Positive Rate
FNR	False Negative Rate
